# Settings for non-household transmission of SARS-CoV-2 during the second lockdown in England and Wales – analysis of the Virus Watch household community cohort study

**DOI:** 10.12688/wellcomeopenres.17981.1

**Published:** 2022-08-03

**Authors:** Susan Hoskins, Sarah Beale, Vincent Nguyen, Ellen Fragaszy, Annalan M.D. Navaratnam, Colette Smith, Clare French, Jana Kovar, Thomas Byrne, Wing Lam Erica Fong, Cyril Geismar, Parth Patel, Alexei Yavlinksy, Anne M. Johnson, Robert W. Aldridge, Andrew Hayward, Virus Watch Collaborative

**Affiliations:** 1 Centre for Public Health Data Science, Institute of Health Informatics, University College London, London, Greater London, WC1E 6BT, UK; 2 Institute of Epidemiology and Healthcare, University College London, London, Greater London, WC1E 7HB, UK; 3 Department of Infectious Disease Epidemiology, London School of Hygiene & Tropical Medicine, London, Greater London, WC1E 7HT, UK; 4 NIHR Health Protection Research Unit in Behavioural Science and Evaluation, Uinversity of Bristol, Bristol, BS8 2BN, UK; 5 Institute for Global Health, University College London, London, WC1N 1EH, UK

**Keywords:** SARS-CoV-2, Covid-19, transmission, shopping, public transport, work, activities, pandemic, lockdown

## Abstract

**Background**: “Lockdowns” to control serious respiratory virus pandemics were widely used during the coronavirus disease 2019 (COVID-19) pandemic.  However, there is limited information to understand the settings in which most transmission occurs during lockdowns, to support refinement of similar policies for future pandemics.

**Methods**: Among Virus Watch household cohort participants we identified those infected with severe acute respiratory syndrome coronavirus 2 (SARS-CoV-2) outside the household.  Using survey activity data, we undertook multivariable logistic regressions assessing the contribution of activities on non-household infection risk.  We calculated adjusted population attributable fractions (APAF) to estimate which activity accounted for the greatest proportion of non-household infections during the pandemic’s second wave.

**Results**: Among 10,858 adults, 18% of cases were likely due to household transmission.  Among 10,475 participants (household-acquired cases excluded), including 874 non-household-acquired infections, infection was associated with: leaving home for work or education (AOR 1.20 (1.02 – 1.42), APAF 6.9%); public transport (more than once per week AOR 1.82 (1.49 – 2.23), public transport APAF 12.42%); and shopping (more than once per week AOR 1.69 (1.29 – 2.21), shopping APAF 34.56%).  Other non-household activities were rare and not significantly associated with infection.

**Conclusions: **During lockdown, going to work and using public or shared transport independently increased infection risk, however only a minority did these activities.  Most participants visited shops, accounting for one-third of non-household transmission.  Transmission in restricted hospitality and leisure settings was minimal suggesting these restrictions were effective.   If future respiratory infection pandemics emerge these findings highlight the value of working from home, using forms of transport that minimise exposure to others, minimising exposure to shops and restricting non-essential activities.

## Introduction

The severe acute respiratory syndrome coronavirus 2 (SARS-CoV-2) pandemic has shown that despite the ability to develop, evaluate and roll out diagnostics, treatments and vaccines (DTVs) rapidly, non-pharmaceutical interventions (NPIs) are a critical component of control of respiratory infection pandemics, particularly prior to widespread vaccination coverage. The pandemic preparedness partnership of the UK Cabinet Office, which has set itself the goal of having DTVs ready within 100 days of a future pathogen being sequenced, asks us to “imagine a scenario where COVID-19 had hit, but the world was ready” ^1^. In order to be ‘ready’ for the next pandemic, we must learn from this one, including developing a better understanding of the effectiveness of NPIs ^2^.

At the beginning of a pandemic in the absence of DTVs, public health measures, NPIs, are relied upon to slow the spread of infections ^3^. When SARS-CoV-2 was first identified, governments introduced mandatory ‘lockdowns’ (Stay at home orders) to reduce direct and close contact between people ^4^. Lockdowns are the most disruptive forms of such NPIs and were widely used internationally for the first time during the SARS-CoV-2 pandemic. These lockdowns enforced intense restrictions across the whole of society: they included advice to work from home where possible, travel was restricted, non-essential businesses such as hospitality and leisure venues were closed, social gatherings that could act as superspreader events were cancelled, mixing socially was restricted and social distancing measures were put in place ^3, 5– 7^. The effects of intense social restrictions have been far reaching, with delays in access to other health care services, mental health and economic consequences some of the many well documented adverse outcomes. While city lockdowns have been shown to be effective in reducing the spread of SARS-CoV2 ^8, 9^, there is limited understanding of settings where transmission continued to occur during these periods of intense societal restrictions. This information is needed to refine such policies to respond more effectively to future pandemic threats.

Understanding which specific restrictions are most effective requires an appreciation of which activities contribute most to viral transmissions but this remains poorly understood ^10^. A review of empirical studies from the first wave of the global health emergency suggests that early school, workplace and business closures and banning of public events had the greatest impact on transmission ^11^. However, first-wave data were limited as national testing capacity was low and infections data were incomplete and inferences were often drawn from ecological studies using population level data. Assessing the relative contribution of different but related activities such as work, transport use and shopping requires collection of individual level data both on infections and activities to untangle these risks ^12– 15^. Three cohort studies have identified community risk factors for SARS-CoV-2 transmission (two from the USA and one pooling data from 99 countries): having an increased number of non-household contacts (OR 1.10 per 10 contacts), air travel (IRR 1.52), employment (IRR 2.50), shopping (IRR 10. 57), frequency of attending events of at least 10 people (OR 1.26 per 10 events), participating in more than one non-essential activity per day, attending various indoor settings (IRR 1.94) including restaurant visits (OR 1.95 per 10 visits, or IRR 1.93), places of worship (IRR 1.92), or gyms or salons (IRR 3.23) ^16– 18^. The largest of these studies used data from early on in the pandemic when community testing was limited and as such their findings may have underestimated incidence (identifying cases among 0.4% of participants) and thus not been able to capture the effect of certain settings and activities. Furthermore, none of the reported study populations were restricted to non-household cases.

By the time of the second wave (September 2020 – end April 2021) of the UK epidemic, which was largely spent under periods of national and regional restrictions, the country had scaled-up access to free polymerase chain reaction (PCR) testing and at-home lateral-flow tests and antibody tests had been developed allowing better data on case ascertainment ^19^. We aimed to understand the relative importance of different activities and settings in the transmission of SARS-CoV-2 in England and Wales during the second wave of the UK epidemic.

## Methods

### Study setting

The analyses are based on the Virus Watch Community Cohort, a study approved by the Hampstead NHS Health Research Authority Ethics Committee. Ethics approval number - 20/HRA/2320, and the detailed methodology of which is described elsewhere ^20^. Briefly, the study recruits whole households with detailed baseline information, weekly surveys of symptoms and self-reported positive SARS-CoV-2 tests (PCR or lateral flow) conducted through the national tracing programme, linkage to the national testing data-set, and monthly questionnaires on contact and activity patterns. A subset of the cohort was invited for antibody testing. Antibody testing was undertaken from October 2020 through venous blood draws in clinics who are part of the National Institute for Health Research’s Clinical Research Network and from March 2021 through monthly finger-prick testing when samples were self-collected by participants using an at-home capillary blood sample collection kit, manufactured by the company Thriva. Completed kits were returned to UKAS-accredited laboratories for serological testing using Roche’s Elecsys Anti-SARS-CoV-2 assays targeting total immunoglobulin (Ig) to the Nucleocapsid (N) protein or to the receptor-binding domain in the S1 subunit of the Spike protein (S).

### Study participants

We identified a sub-cohort of Virus Watch participants who completed two monthly behavioural surveys during the second wave (completed during 1/12/2020 – 10/12/2020 and 17/02/2021 – 28/02/2021). We restricted to those in the antibody testing cohort to ensure that infection outcomes could be ascertained in all participants regardless of their swabbing behaviour. 

We included cases who were infected with SARS-CoV-2 during the second wave of the UK epidemic: anyone testing PCR or lateral flow positive between 01/10/2020 and 01/05/2021, or anyone who tested positive for nucleocapsid antibody due to likely infection versus vaccination between 01/10/2020 and 01/05/2021 unless they had previously reported a positive PCR or lateral flow test or previously tested positive for antibodies on venous blood samples was considered to have been infected in the second wave of the pandemic. 

In order to focus analyses on risk factors for non-household transmission (defined as cases acquired outside the home) we further restricted cases to those which were the only case in the household (no other antibody or PCR or lateral flow cases in the household) and, in households with more than one PCR/lateral flow confirmed case, to i) the first case in the household based on the earliest date of symptom onset in the house, or ii) where there were multiple positive people in the household reporting the same symptom onset date, all cases were included as co-primaries, or iii) if no symptom data were available, based on the earliest date of PCR or lateral flow test in the house using dates from the National Testing data, or if National testing date data were not available, using the Virus Watch given date or imputed middle of the week date when the test was self-reported. Where we could not identify who was the likely first case in the household these infections were excluded.

### Outcome variable

SARS-CoV-2 infection was defined as having any of i) a positive self-reported PCR test, ii) a positive self-reported lateral flow test, iii) a positive PCR or lateral flow test from data linkage to the National Testing data, iv) seropositivity to the Nucleocapsid protein (COI ≥ 1.0) among vaccinated individuals, or seropositivity to either Spike (≥0.8 U/ml) or Nucleocapsid protein among unvaccinated individuals. Samples with void N-Ab or void S-Ab results were excluded from analyses. The primary source of data was the Virus Watch dataset. To maximise data on cases, we linked to the Second Generation Surveillance System (SGSS) which contains SARS-CoV-2 results using data from hospitalisations (Pillar 1) and community testing (pillar 2). Linkage was conducted by NHS Digital with the linkage variables were sent on the 19 ^th^ of March 2021. The linkage period encompassed data from March 2020 until August 2021.

### Exposure variables

From the monthly behavioural surveys, we examined the frequency of leaving home to go to work or education, using public or shared transport, going to retail settings and visiting other non-household settings in the week prior to the survey. Participants also provided information on the number of known close contacts outside the household during the week before each survey, where close contact was defined as being within 2m of someone for more than 15 minutes.

### Statistical methods

The weekly frequency of activities and number of close contacts was averaged over the two surveys to give an average weekly frequency of activities and average number of contacts, as a proxy for activity and contact patterns during the second wave of the pandemic. We created composite variables for public or shared transport activities (use of taxi or shared car, bus, over and underground rail and air travel), retail activities (use of essential and non-essential shops and outdoor markets) and other activities undertaken (all other non-transport, non-work, non-retail activities including going to the theatre, cinema, a concert or an outdoor sports event, going to a bar, pub, club or disco, eating in a restaurant, café or canteen, attending a party, going to a place of worship, going to a gym or participating in indoor sport, and going to a hairdresser, barber, beautician or a nail salon). We undertook univariable analyses comparing the proportion with evidence of infection acquired outside the household, according to the weekly frequency of the individual activity exposures and univariable analyses according to the weekly frequency of the composite exposures including going to work, using public or shared transport, using retail venues and other non-household contacts. For the composite exposures, we conducted a multivariable regression model incorporating all composite exposures and demographic variables found to have been associated at the univariable level, and we included age to the model on the basis that age is significantly related to activity levels. We hypothesised that the risk of infection acquired in non-household settings is, at least in part, mediated by the number of known close contacts outside the household so we have not controlled for this in the main analysis but have conducted a sensitivity analysis additionally adjusting for number of known contacts outside the home to gain insight how transmission in different settings may be mediated by known close contact or more distant, shorter or unrecognised contact. We undertook an additional adjusted model for the composite exposures which did not control for public/shared transport use as it may be on the causal pathway between work and infection. Analyses were carried out using STATA version 16 (RRID:SCR_012763). 

For the composite variables with multiple levels of exposure, we calculated stratum specific (up to once a week and more than once a week for transport use and retail, and under twice a week, two- three times a week, more than three to five times a week, and more than 5 times a week for other activities) and total adjusted multivariate population attributable fractions (aPAF). We calculated aPAF (the proportion of non-household transmission in the cohort thought to be attributable to each exposure) based on odds ratios and frequency of exposure in cases. We used the formula PAF = p * (1-1/relative risk) where p=proportion of those with SARS-CoV-2 acquired outside the household who had the exposure of interest and where the adjusted odds ratios were taken as proxies of relative risk (based on the rare outcome assumption) to calculate adjusted PAFs. Stratum specific and total PAFs were calculated using Microsoft Excel version 13. Missing data were sparse and while included in the univariate analyses, participants with missing data were not included in the multivariate adjusted models and do not contribute to the PAFs.

## Results

Of the 10,858 participants who completed the two monthly surveys and were members of the antibody cohort, 1,257 (12%) were identified as having had SARS-CoV-2 (343 were identified by either PCR or lateral flow test and 914 by likely infection-induced antibody results alone). Of all cases identified via PCR or lateral flow test, we estimate up to 18% of these may be due to household transmission based on the timing of illnesses and swabs in other household members ( Table 1). 

**Table 1.** Proportion of overall infection attributable to household and non-household transmission.

Identification of primary, co-primary and secondary cases	Number in cohort (n=10,858)	Proportion of cases (%)
A) Number with no evidence of infection	9601	
Number of positive Polymerase Chain Reaction (PCR)/Lateral Flow tests	343	
b) Secondary cases (no symptom data but someone in the house tests (+) first, by National Testing. If national testing data date was not available then the date of onset was imputed as the middle of the week when the participant reported they tested positive) (% of PCR/Lateral flow cases)	16	5%
a) Secondary case (someone else in home who is PCR/Lateral Flow positive has symptoms prior to case) (% of PCR/Lateral flow cases)	47	14%
Upper Limit Household transmission (number and proportion of PCR/Lateral Flow confirmed cases that are thought to be secondary cases a+b/a+b+c+d+e)	63	18%
c) Primary case: only case in a home (% of PCR/Lateral flow cases)	189	55%
d) Co-primary case: symptom date the same as earliest symptom in the home (% of PCR/Lateral flow cases)	85	25%
e) Primary case: no symptom data but tests first in the home by National Testing data. If National testing data not available, by VW imputed mid-week date (% of PCR/Lateral flow cases)	6	2%
Non-household transmission (number and proportion of PCR/Lateral Flow confirmed cases that are thought to be due to non-household transmission (c+d+e/a+b+c+d+e)	280	82%
Number of Antibody (AB) (+) PCR (-) cases	914	
f) Primary AB case: only AB (+) PCR (-) case in the home (i.e. no other AB (+) or PCR (+) case in the home) (% of AB (+) PCR (-) cases)	594	65%
g) Potential secondary case: AB (+) PCR (-) case with someone else infected in the home either as - another AB (+) PCR (-) case in the home or - a (+) PCR case in the home Total potential secondary AB (+) PCR (-) (proportion of AB (+) PCR (-) cases)	279 41 320	35%
Likely cases of secondary infection (a+b+g)	383	
Likely first/only case in the home (c+d+e+f)	874	
Non-household transmission cohort (c+d+e+f+A)	10,475	

The non-household transmission cohort which formed these analyses, therefore included 10475 participants; 874 (8%) SARS-COV-2 cases identified through PCR, lateral flow and/or positive antibody tests who were thought to have been infected outside the household during the second wave of the pandemic and 9601 uninfected participants ( Table 1). The breakdown of the cohort by age, gender, index of multiple deprivation, region, ethnicity and vaccination status are shown in Table 2 with univariate analyses of the risk of infection acquired outside the household in each group. The highest levels of infection were seen in working-aged adults (12%), women (10%), poorer areas (11%), non-white ethnic groups (up to 13%), London (14%) and the unvaccinated (14%).

**Table 2.** Participant characteristics and risk of infection acquired outside the household during the pandemic second wave.

Characteristic	Category	Number in cohort N=10,475	Proportion in category (%)	Number of cases n=874	Proportion in category (%)	Unadjusted Odds Ratio	95% CI	P
Vaccine status	Yes No	9,502 973	91% 9%	736 138	8% 14%	1.00 1.97	1.62 – 2.39	<0.0001
Age	Under 18 Working Age 65 and above Missing	383 5,412 4,669 11	4% 52% 45%	23 634 217	6% 12% 5%	1.00 2.08 0.76	1.35 – 3.19 0.49 – 1.19	<0.0001
Sex	Male Female Missing	4,538 5,913 24	43% 57%	308 563	7% 10%	1.00 1.44	1.25 – 1.67	<0.0001
Ethnic group	White White Other Asian Black Mixed Other Missing	9,475 589 240 55 59 49 8	91% 6% 2% 1% 1% <1%	774 54 31 5 6 4	8% 9% 13% 9% 10% 8%	1.00 1.13 1.67 1.12 1.27 0.99	0.85 – 1.52 1.14 – 2.45 0.45 – 2.83 0.55 – 2.97 0.36 – 2.79	0.2302
Deprivation score (IMD quintile) 1= most deprived	1 2 3 4 5 Missing	778 1,538 2,099 2,815 3,230 15	7% 15% 20% 27% 31%	71 165 177 225 236	9% 11% 8% 8% 7%	1.27 1.52 1.17 1.10 1.00	0.97 – 1.68 1.24 – 1.88 0.95 – 1.43 0.91 – 1.33	0.0026
Region	East Midlands East of England London North East North West South East South West Wales West Midlands Yorkshire & The Humber Missing	1,067 2,236 1,125 554 1,233 1,840 948 255 588 614 15	10% 21% 11% 5% 12% 18% 9% 2% 6% 6%	83 160 159 51 96 146 54 24 49 52	8% 7% 14% 9% 8% 8% 6% 9% 8% 8%	1.00 0.91 1.95 1.20 1.00 1.02 0.72 1.23 1.08 1.09	0.69 – 1.20 1.48 – 2.58 0.83 – 1.73 0.74 – 1.36 0.77 – 1.35 0.50 – 1.02 0.76 – 1.98 0.75 – 1.56 0.76 – 1.58	<0.0001

*Missings are not included in the percentage

Table 3 shows the detailed breakdown of public or shared transport exposures in relation to risk of infection. On univariable analysis, all forms of public or shared transport, with the exception of using an airplane, which was rare, were associated with an increased risk of SARS-CoV-2 infection. Table 4 shows the detailed breakdown of out of household close contact and activities other than work and public/shared transport use and associations with non-household acquired infections. The only significantly associated activity during this period was with essential retail, although exposure to other settings such as leisure and hospitality was rare. Having an increasing number of close contacts outside the home compared to having none was associated with infection (p<0.0001): up to two close contacts OR 1.09 (0.91- 1.29), more than two to five close contacts OR 1.57 (1.27 – 1.95), more than five to ten close contacts OR 2.12 (1.57 – 2.87), more than 10 close contacts OR 1.99 (1.53 – 2.58).

**Table 3.** Risk of infection according to type and frequency of public or shared transport use

Activity undertaken	Weekly frequency	Number in cohort (N=10,475)	Proportion in cohort (%)	Covid infection (N=874)	Proportion of cases in cohort (%)	Univariate Odds Ratio	95% CI	p
Used a taxi or car shared with someone outside the household	No Yes	8,395 2,080	80% 20%	638 236	8% 11%	1.00 1.56	1.33 – 1.82	<0.0001
Used a bus	0 >0 – 2 >2	9,708 604 163	93% 6% 2%	766 86 22	8% 14% 14%	1.00 1.94 1.82	1.52 – 2.46 1.16 – 2.87	0.0001
Used an over-ground train or tram	0 >0 – 2 >2	10,099 307 69	96% 3% 1%	814 48 12	8% 16% 17%	1.00 2.11 2.40	1.54 – 2.90 1.28 – 4.49	<0.0001
Used an underground train	0 >0 – 2 >2	10,172 251 52	97% 2% 1%	812 54 8	8% 22% 15%	1.00 3.16 2.09	2.32 – 4.31 0.98 – 4.47	<0.0001
Used an airplane	No Yes	10,401 74	99% 1%	864 10	8% 14%	1.00 1.72	0.88 – 3.37	0.1353

**Table 4.** Risk of infection according to frequency of non-work or education and non-public or shared transport activities outside the household.

Activities	Weekly frequency	Number in cohort (N=10,475)	Proportion in cohort (%)	Covid infection (N=874)	Proportion of cases in cohort (%)	Univariate Odds Ratio	95% CI	p
Played a team sport outdoors	No Yes	10,319 156	99% 1%	862 12	8% 8%	1.00 0.91	0.51– 1.65	0.7642
Went to a theatre, cinema, concert or sports event	No Yes	10,430 45	99% 1%	868 6	8% 13%	1.00 1.69	0.72 – 4.01	0.2602
Went to a shop for essential items	0 >0 – 2 >2 – 4 >4 – 5 >5	1,748 6,273 2,063 219 172	17% 60% 20% 2% 2%	90 541 202 24 17	5% 9% 10% 11% 10%	1.00 1.74 1.99 2.27 2.02	1.38 – 2.19 1.55 – 2.59 1.41 – 3.64 1.17 – 3.48	<0.0001
Went to a shop for non-essential items	No Yes	8,187 2,288	78% 22%	677 197	8% 9%	1.00 1.05	0.89 – 1.23	0.6034
Went to a bar, pub, club, disco	No Yes	10,361 114	99% 1%	864 10	8% 9%	1.00 1.06	0.55 – 2.03	0.8689
Ate at a restaurant, café or canteen	No Yes	10,100 375	96% 4%	834 40	8% 11%	1.00 1.33	0.95 – 1.86	0.1104
Went to a party	No Yes	10,446 29	99% <1%	872 2	8% 7%	1.00 0.81	0.19 – 3.43	0.7717
Went to a place of worship	No Yes	10,124 351	97% 3%	849 25	8% 7%	1.00 0.84	0.55 – 1.27	0.3894
Went to an outdoor market	No Yes	9,806 669	94% 6%	816 58	8% 9%	1.00 1.04	0.79 – 1.38	0.7540
Went to a gym/indoor sport	No Yes	10,284 191	98% 2%	858 16	8% 8%	1.00 1.00	0.59 – 1.68	0.9866
Went to a hairdresser, barber, nail salon, beauty parlour	No Yes	10,212 263	97% 3%	853 21	8% 8%	1.00 0.95	0.61 – 1.49	0.8301
Number of close contacts outside the home	0 >0 – 2 >2 – 5 > 5 – 10 >10	3,294 4,553 1,482 448 698	31% 43% 14% 4% 7%	228 340 155 61 90	7% 7% 10% 14% 13%	1.00 1.09 1.57 2.12 1.99	0.91 – 1.29 1.27 – 1.95 1.57 – 2.87 1.54 – 2.58	<0.0001

Table 5 shows the relative impact of leaving home to go to work or education, using public or shared transport, retail and other non-household activities on the risk of infection acquired outside the household. Models are adjusted for all variables in the table in addition to age, gender, ethnicity, region, vaccination and index of multiple deprivation quintile. Table 5 also shows the unadjusted (raw) and adjusted population attributable fractions for leaving home for work, public or shared transport and retail.

**Table 5.** Unadjusted and adjusted odds ratios and population attributable fractions for non-household SARS-CoV-2 infection.

Activity	Weekly frequency	Number in cohort (n=10,475)	Proportion in cohort (%)	Number of cases (n=874)	Proportion of cases in cohort (%)	Odds Ratio (95% CI), p	Adjusted Odds Ratio (95% CI), p	Population attributable fraction (%) Raw (adjusted)
Leaving home for work or education	No Yes	7,266 3,209	69% 31%	507 367	7% 11%	1 1.72 (1.49 – 1.98) p<0.001	1.00 1.20 (1.02 – 1.42) P=0.0307	17.58 (Adj-6.99)
Weekly frequency of using public or shared transport	0 >0 -1 >1	7,621 1,733 1,121	73% 17% 11%	539 165 170	7% 10% 15%	1.00 1.38 (1.15 – 1.66) 2.35 (1.95 – 2.83) P<0.001	1.00 1.24 (1.03 – 1.49) 1.82 (1.49 – 2.23) p<0.0001	5.19 (Adj-3.65) 11.17(Adj-8.76) Total 16.37 (Adj 12.42)
Weekly frequency of any retail	0 >0 -1 >1	1,560 3,030 5,885	15% 29% 56%	78 234 562	5% 8% 10%	1.00 1.59 (1.22 – 2.07) 2.01 (1.57 – 2.56) P<0.001	1.00 1.45 (1.09 – 1.92) 1.69 (1.29 – 2.21) P=0.0003	9.93 (Adj-8.31) 32.31 (Adj-26.25) Total 42.25 (Adj 34.56)
Weekly frequency of other non- household activities	0 >0 – <2 2 – 3 >3 – 5 > 5	823 2,454 2,396 2,620 2,182	8% 23% 23% 25% 21%	51 184 191 248 200	6% 8% 8% 9% 9%	1.00 1.23 (0.89 – 1.69) 1.31 (0.95 – 1.81) 1.58 (1.16 – 2.16) 1.53 (1.11 – 2.10) P<0.0066	1.00 0.99 (0.71 – 1.39) 0.88 (0.63 – 1.23) 0.97 (0.69 – 1.36) 0.87 (0.61 – 1.23) P=0.6188	3.94 5.17 10.41 7.93 Total 27.45 (Adjusted not estimated)

The odds ratio for leaving home for work or education at least once per week was 1.72 but this association was considerably weakened after controlling for public transport use and other risk factors, adjusted OR 1.20 (1.02 – 1.42) p=0.0307. Across the cohort leaving home for work accounted for 18% of non-household acquired infections (PAF) but this was reduced to 7% after controlling for transport use and other variables ( Table 5). In the additional adjusted model (not shown) which did not control for public or shared transport use leaving home for work was associated with an increased odds of adjusted OR 1.29 (CI 1.09 – 1.52, P=0.0026).

The odds ratio for using public or shared transport more than once per week compared to no usage was 2.35 but this was reduced after controlling for going to work or education and other risk factors (adjusted OR 1.82 (1.49 – 2.23) p<0.0001) ( Table 5). Across the cohort, public or shared transport use accounted for 16% of infections acquired outside the household, though this was reduced to 12% after controlling for going to work and other variables.

The odds ratio for using shops more than once per week compared to no usage was 2.01 but reduced after controlling for other variables (adjusted OR 1.69 (1.29 – 2.21) P=0.0003). Across the cohort, shopping accounted for 42% of infections acquired outside the household and 35% after controlling for other variables ( Table 5).

Table 6 and Table 7 show the unadjusted and adjusted odds ratios and PAFs for infection acquired outside the household in the working-age population (18–64) and in those aged > 64 years, respectively. Of note, among those of working age, leaving home for work or education and using public or shared transport each accounted for around 10% of non-household acquired infections (adjusted PAFs 8.99% and 10.94%, respectively) while using shops accounted for 32% (adjusted PAF 31.87%). For those aged 65 years and above, using public or shared transport (adjusted PAF 14.06%) and shopping (adjusted PAF 38.41%) accounted for the greatest proportion of non-household acquired infections. Leaving home for work was rare in this age group and not significantly associated with risk of infection. 

**Table 6.** Unadjusted and adjusted odds ratios and population attributable fractions for non-household SARS-CoV-2 infection among those of working age (18 – 64 years).

Activity	Weekly frequency	Number in cohort (N=5,412)	Proportion in cohort (%)	Number of cases (n=634)	Proportion of cases in cohort (%)	Odds Ratio (95% CI), p	Adjusted Odds Ratio (95% CI), p	Population Attributable Fraction (%) Raw (adjusted)
Leaving home for work or education	No Yes	3,008 2,404	56% 44%	318 316	11% 13%	1 1.28 (1.08 – 1.51) P=0.0035	1.00 1.22 (1.02 – 1.47) P=0.0329	10.90 (Adj 8.99)
Weekly frequency of using public or shared transport	0 >0 -1 >1	3,850 900 662	71% 17% 12%	396 117 121	10% 13% 18%	1.00 1.30 (1.04 – 1.62) 1.95 (1.56 – 2.44) P<0.001	1.00 1.19 (0.96 – 1.50) 1.72 (1.35 – 2.19) P=0.0001	4.26 (Adj 2.95) 9.29 (Adj 7.99) Total 13.56 (Adj 10.94)
Weekly frequency of any retail	0 >0 - 1 >1	636 1,540 3,236	12% 28% 60%	50 169 415	8% 11% 13%	1.00 1.44 (1.04 – 2.01) 1.72 (1.27 – 2.34) P=0.0006	1.00 1.38 (0.99– 1.94) 1.60 (1.16 – 2.22) P=0.0097	8.14 (Adj 7.34) 27.4 (Adj 24.55) Total 35.55 (Adj 31.87)
Weekly frequency of other non household activities	0 >0 – <2 2 – 3 >3 – 5 > 5	349 1,073 1,210 1,457 1,323	6% 20% 22% 27% 24%	37 125 136 171 165	11% 12% 11% 12% >12%	1.00 1.11 (0.75 – 1.64) 1.07 (0.73 – 1.57) 1.12 (0.77 – 1.63) 1.20 (0.82 – 1.75) P=0.8404	1.00 0.91 (0.61 – 1.35) 0.78 (0.52 – 1.16) 0.77 (0.51 – 1.15) 0.79 (0.53 – 1.21) P=0.5716	1.95 1.40 2.89 4.34 Total 10.58 Adjusted not estimated

**Table 7.** Unadjusted and adjusted odds ratios and population attributable fractions for non-household SARS-CoV-2 infection among those of retired age (65 years and above).

Activity	Weekly frequency	Number in cohort (N=4,669)	Proportion in cohort (%)	Number of cases (n=217)	Proportion of cases in cohort (%)	Odds Ratio (95% CI), p	Adjusted Odds Ratio (95% CI), p	Population Attributable Fraction (%) Raw (adjusted)
Leaving home for work or education	No Yes	4,220 449	90% 10%	189 28	4% 6%	1 1.42 (0.94 – 2.14) P=0.1073	1.00 1.28 (0.82 – 1.99) P=0.2806	3.82 (Adj 2.82)
Weekly frequency of using public or shared transport	0 >0 -1 >1	3,515 795 359	75% 17% 8%	132 46 39	4% 6% 11%	1.00 1.57 (1.11 – 2.22) 3.12 (2.15 – 4.54) P<0.001	1.00 1.29 (0.89 – 1.87) 2.07 (1.36 – 3.14) P=0.0042	7.69 (Adj 4.77) 12.21 (Adj 9.29) Total 19.91 (Adj 14.06)
Weekly frequency of any retail	0 >0 -1 >1	669 1,400 2,600	14% 30% 56%	17 57 143	3% 4% 6%	1.00 1.62 (0.94 – 2.82) 2.23 (1.34 – 3.72) P=0.0014	1.00 1.59 (0.89 – 2.83) 1.77 (1.01 – 3.09) P=0.1083	10.05 (Adj 9.75) 36.35 (Adj 28.67) Total 46.40 (Adj 38.41)
Weekly frequency of other non household activities	0 >0 – <2 2 – 3 >3 – 5 > 5	434 1,340 1,050 1,048 797	9% 29% 22% 22% 17%	14 57 49 66 31	3% 4% 5% 6% 4%	1.00 1.33 (0.74 – 2.42) 1.47 (0.80 – 2.69) 2.02 (1.12 – 3.63) 1.21 (0.64 – 2.31) P=0.0477	1.00 1.12 (0.59 – 2.12) 1.07 (0.55 – 2.07) 1.47 (0.77 – 2.80) 0.88 (0.43 – 1.78) P=0.2195	6.52 (Adj 2.81) 7.22 (Adj 1.48) 15.36 (Adj 9.72) 2.48 (Adj -1.95) Total 31.57 (Adj12.07)

Table 8 shows the effect of leaving home for work, public or shared transport use and shopping after additionally controlling for the number of close contacts outside the home and the previously described adjustment variables. The remaining risk from attending work is further ameliorated (adjusted OR 1.10 95% CI 0.92 – 1.32 p= 0.30). Adjusted odds ratios for public or shared transport and shopping were minimally affected by adding the number of non-household close contacts as a covariate. Notably, despite significant univariate associations between ethnicity and social deprivation and COVID risk, the full final multivariate model ( Table 8) showed no significant difference in risk of acquiring SARS-CoV-2 outside the household by ethnicity (p= 0.12) or social deprivation quintile (p=0.71) after controlling for confounders including work, public or shared transport use, shopping and other variables.

**Table 8.** Multivariate analysis of number of contacts outside household, non-household activities and risk of infection (all variables are mutually adjusted).

Activities	Weekly frequency	Adjusted OR	(CI)	p
Number of contacts outside the home	0 >0 - 2 >2 - 5 > 5 - 10 >10	1.00 0.93 1.15 1.34 1.35	0.78 – 1.12 0.91 – 1.45 0.96 – 1.87 0.99 – 1.83	0.0431
Leaving home for work or education	No Yes	1.00 1.10	0.92 – 1.32	0.2967
Weekly frequency of using public or shared transport	0 >0 -1 >1	1.00 1.23 1.76	1.01 – 1.49 1.43 – 2.16	<0.0001
Weekly frequency of any retail	0 >0 -1 >1	1.00 1.46 1.69	1.10 – 1.93 1.29 – 2.22	0.0003
Weekly frequency of other activities	0 >0 - >1 2 – 3 >3 – 5 > 5	1.00 1.00 0.87 0.95 0.83	0.72 – 1.39 0.62 – 1.22 0.68 – 1.34 0.59 – 1.19	0.4742
Vaccine status	Yes No	1.00 1.99	1.59 – 2.49	<0.0001
Age	Under 18 Working Age 65 and above	1.00 3.60 1.61	2.17 – 5.97 0.94 – 2.76	<0.001 0.082
Ethnic group	White White Other Asian Black Mixed Other	1.00 0.67 0.97 0.66 0.69 0.55	0.49 – 0.92 0.64 – 1.46 0.26 – 1.69 0.29 – 1.66 0.19 – 1.59	0.1196
Deprivation score (IMD quintile) 1= most deprived	1 2 3 4 5	0.92 1.13 1.02 1.02 1.00	0.69 – 1.23 0.91 – 1.42 0.83 – 1.26 0.84 – 1.24	0.7067
Region	East Midlands East of England London North East North West South East South West Wales West Midlands Yorkshire & The Humber	1.00 0.96 1.62 1.17 0.99 1.04 0.77 1.27 1.12 1.19	0.73 – 1.28 1.19 – 2.19 0.80 – 1.70 0.72 – 1.35 0.78 – 1.39 0.54 – 1.10 0.78 – 2.07 0.76 – 1.62 0.82 – 1.72	0.0027
Sex	Male Female	1.00 1.36	1.17 – 1.58	<0.001

## Discussion

The study demonstrates that leaving home for work or education, using public or shared transport and shopping were important independent risk factors for acquiring SARS-CoV-2 outside the household during the second wave of the pandemic in England and Wales, a period of intense restrictions on mixing. Although those going to work had a substantially higher risk of infection much of this was explained by public or shared transport use and other variables. This suggests that part of the risk associated with going to work or education was due to exposure on public or shared transport. Public or shared transport use remained a strong independent risk factor after controlling for other variables. Shopping was also an important risk factor for acquiring SARS-CoV-2 outside the household and, because it was a very common exposure, accounted for a high proportion of infections acquired outside the household in adults. Other non-household activities such as visiting hospitality or leisure venues were not significantly associated with acquiring SARS-CoV-2 outside the household but were rare during this period of intense restrictions. The risk of infection acquired outside the household was strongly associated with the number of close contacts outside the household. Controlling for this further ameliorated the effect of attending work suggesting this is in part mediated by close contact at work. Controlling for close contact made little difference to associations with public or shared transport and shopping suggesting these exposures are not mediated by recognized close contact and may represent more distant aerosol-based transmission or unrecognised close contact ^21^. It was interesting that the effect of ethnicity and social deprivation was not seen after accounting for work, public or shared transport use, shopping and other variables – suggesting different patterns of exposure to work, associated public or shared transport use and use of shops may account for differential infection rates.

Our findings, which demonstrate increased risk of infection with increased levels of activity, are similar to those found through other studies. The COVID-19 Citizen Science Study, which gathered data from participants across 99 countries, found an increased risk of infection with the number of non-household contacts (OR 1.10 per 10 contacts) ^16^. Mehta *et al*., when estimating community risk of infection around the Thanksgiving holiday period, found that participating in more than one non-essential activity per day was associated with an increased risk of infection ^18^. And among a national prospective cohort of 4,510 adults in the USA, public transport (air travel) (IRR 1.52) was seen to increase risk of infection ^17^.

By restricting our analysis to those in the cohort with antibody test results we could ensure that infection could be ascertained in all participants regardless of their testing behaviour (although antibody waning may lead to loss of detectable nucleocapsid antibody). Due to limited testing capacity nationwide during the first wave of the pandemic, it is possible that we have included some individuals with positive antibodies as a result of infection prior to October 2020. By excluding those with positive PCR or lateral flow or antibody tests prior to October 2020, we sought to minimise this potential over-ascertainment bias. While we sought to identify cases who were the first or only case of SARS-CoV-2 in the household it is possible that there was some misclassification error because of uncertainties in the timing of infection and failure to identify all infections in a household. Activities and behaviours are self-reported and therefore subject to recall bias and social desirability bias although data examined during the first wave of the pandemic in Germany were found to support the use of self-reported contact survey data to reflect infection dynamics ^22^. We tried to minimise recall bias by asking about activities in the previous seven days. These activities were sampled at two points during the second wave and may, however, not be representative of the activities throughout the second wave. 

Both self-reported and linked data on test results from the national testing system also allowed ascertainment of infections. Maximising ascertainment of SARS-CoV-2 infections supports accurate assessment of the relative importance of risk factors. A further strength was the household structure of the cohort allowing us to focus analyses onto risk factors for non-household transmission and largely eliminate confounding effects that act on household transmission, although misclassification will have occurred. Outcomes and exposures were both measured during the same wave of the pandemic, although for those with antibody results only it was not possible to ascertain with certainty whether they were infected during the first or the second wave of the pandemic. Population attributable fractions are influenced by the frequency of exposures within the cohort which may not be representative of the entire adult population. For example, if our cohort includes fewer people going to work than on average, then this will lead to underestimation of the PAF related to going to work.

The research suggests that working from home and consequently avoiding the need to use public or shared transport has a significant impact on risk at individual and population level. We could not ascertain the risk associated with transmission in hospitality and leisure venues as such exposures were rare, suggesting that regulations restricting their use was effective in reducing transmission. Shopping which remained a common exposure was an important contributor to risk at individual and population levels. Understanding the relative importance of these activities and settings on transmission during periods where countries employ fewer NPIs remains important and will be the subject of future analyses of the Virus Watch cohort.

In the event of future respiratory virus pandemics or future waves of SARS-CoV-2 variants with potential for high severity, increasing the proportion of people who work from home, facilitating active transport such as cycling or walking in those who need to go to work, and enabling people to shop for essential goods online would be expected to make a highly significant impact on transmission and risk of severe disease. Although high vaccination rates reduce the need for intense non pharmaceutical interventions these measures remain important in poorly vaccinated countries and may become important in the event of SARS-CoV-2 resurgence due to waning immunity, emergence of new variants such as the Omicron variants with immune escape or increased severity ^23^. 

## Data availability

### Underlying data and analysis code

We aim to share aggregate data from this project on our website and via a “Findings so far” section on our website. We are sharing individual record-level data on the Office of National Statistics Secure Research Service, and given the sensitive content in our dataset for this study, we cannot release the data at the individual level. Access to use of the data whilst research is being conducted will be managed by the Chief Investigators (ACH and RWA) in accordance with the principles set out in the UK Research and Innovation Guidance on best practice in the management of research data. Data access requests can also be made directly to the Virus Watch chief investigators (ACH or RWA) at the following email address: viruswatch@ucl.ac.uk. The data along with the analysis code used will be provided to approved researchers.

## References

[1] Gov.UK, Cabinet Office of the Government of the United Kingdom: 100 Days Mission to respond to future pandemic threats – A report to the G7 by the pandemic preparedness partnership. 2021; last accessed 06/05/2022. Reference Source

[2] Fisher D Suri S Carson G : What comes next in the COVID-19 pandemic? *Lancet.* 2022;399(10336):P1691–1692. 10.1016/S0140-6736(22)00580-3 35421374 PMC9000913

[3] Looi MK : The BMJ Interview: WHO chief scientist optimistic for a pan-coronavirus vaccine in two years. *BMJ.* 2022;377:o1003. 10.1136/bmj.o1003 35473706

[4] Gov.UK, Cabinet Office of the Government of the United Kingdom: Staying at home and away from others (social distancing). 2020; last accessed 06/05/2022. Reference Source

[5] Meyerowitz EA Richterman A Gandhi RT : Transmission of SARS-CoV-2: A Review of Viral, Host, and Environmental Factors. *Ann Intern Med.* 2021;174(1):69–79. 10.7326/M20-5008 32941052 PMC7505025

[6] Chiesa V Antony G Wismar M : COVID-19 pandemic: health impact of staying at home, social distancing and ‘lockdown’ measures-a systematic review of systematic reviews. *J Public Health (Oxf).* 2021;43(3):e462–e481. 10.1093/pubmed/fdab102 33855434 PMC8083256

[7] Ebrahim SH Ahmed QA Gozzer E : Covid-19 and community mitigation strategies in a pandemic. *BMJ.* 2020;368:m1066. 10.1136/bmj.m1066 32184233

[8] Ayouni I Maatoug J Dhouib W : Effective public health measures to mitigate the spread of COVID-19: a systematic review. *BMC Public Health.* 2021;21(1):1015. 10.1186/s12889-021-11111-1 34051769 PMC8164261

[9] Iezadi S Gholipour K Azami-Aghdash S : Effectiveness of non-pharmaceutical public health interventions against COVID-19: A systematic review and meta-analysis. *PLoS One.* 2021;16(11):e0260371. 10.1371/journal.pone.0260371 34813628 PMC8610259

[10] Mendez-Brito A El Bcheraoui C Pozo-Martin F : Systematic review of empirical studies comparing the effectiveness of non-pharmaceutical interventions against COVID-19. *J Infect.* 2021;83(3):281–293. 10.1016/j.jinf.2021.06.018 34161818 PMC8214911

[11] Gov.UK, Cabinet Office of the Government of the United Kingdom: PHE Transmission group: Factors contributing to risk of SARS-CoV2 transmission associated with various settings.2020; Last accessed 06/05/2022. Reference Source

[12] Nafilyan V Pawelek P Ayoubkhani D : Occupation and COVID-19 mortality in England: a national linked data study of 14.3 million adults. *medRxiv.* [preprint], 2021.05.12.21257123. 10.1101/2021.05.12.21257123 34965981

[13] Gov.UK, Cabinet Office of the Government of the United Kingdom: SAGE Environmental Modelling Group: COVID-19 risk by occupation and workplace. 2021; last accessed 06/05/2022. Reference Source

[14] Gartland N Fishwick D Coleman A : Transmission and control of SARS-CoV-2 on ground public transport: A rapid review of the literature to date.University of Manchester. Preprint.2021. 10.48420/16622974.v1 PMC8894738 35261878

[15] Gov.UK, Cabinet Office of the Government of the United Kingdom: SAGE Environmental Modelling group: Insights on transmission of COVID-19 with a focus on the hospitality, retail and leisure sector. last accessed 06/05/2022. Reference Source

[16] Lin A Vittinghoff E Olgin J : Predictors of incident SARS-CoV-2 infections in an international prospective cohort study. *BMJ Open.* 2021;11(9):e052025. 10.1136/bmjopen-2021-052025 34548363 PMC8457993

[17] Nash D Rane MS Chang M : SARS-CoV-2 incidence and risk factors in a national, community-based prospective cohort of U.S. adults.Version 2. *medRxiv.* Preprint.2021; 2021.02.12.21251659. [revised 2021 Oct 12]. 10.1101/2021.02.12.21251659 33619505 PMC7899475

[18] Mehta SH Clipman SJ Wesolowski A : Holiday gatherings, mobility and SARS-CoV-2 transmission: results from 10 US states following Thanksgiving. *Sci Rep.* 2021;11(1):17328. 10.1038/s41598-021-96779-6 34462499 PMC8405672

[19] Office for National Statistics, Government of the United Kingdom : Coronavirus (COVID-19) Infection Survey technical article: waves and lags of COVID-19 in England. 2021; last accessed 06/05/2022. Reference Source

[20] Hayward A Fragaszy E Kovar J : Risk factors, symptom reporting, healthcare-seeking behaviour and adherence to public health guidance: protocol for Virus Watch, a prospective community cohort study. *BMJ Open.* 2021;11(6):e048042. 10.1136/bmjopen-2020-048042 34162651 PMC8230990

[21] Leung NHL : Transmissibility and transmission of respiratory viruses. *Nat Rev Microbiol.* 2021;19(8):528–545. 10.1038/s41579-021-00535-6 33753932 PMC7982882

[22] Tomori DV Rübsamen N Berger T : Individual social contact data and population mobility data as early markers of SARS-CoV-2 transmission dynamics during the first wave in Germany—an analysis based on the COVIMOD study. *BMC Med.* 2021;19(1):271. 10.1186/s12916-021-02139-6 34649541 PMC8515158

[23] Zhang Y Quigley A Wang Q : Non-pharmaceutical interventions during the roll out of covid-19 vaccines. *BMJ.* 2021;375:n2314. 10.1136/bmj.n2314 34853011 PMC8634371

