# Supplementary material for: Settings for non-household transmission of SARS-CoV-2 during the second lockdown in England and Wales – analysis of the Virus Watch household community cohort study
Source: Wellcome Open Res. 2022 Aug 3;7:199. [Version 1] doi: 10.12688/wellcomeopenres.17981.1 (PMC9975411; doi:10.12688/wellcomeopenres.17981.1)
Supplement: Peer review history

# Peer review: Reviewer response for version 1

**DOI:** 10.21956/wellcomeopenres.19932.r53603

**Published:** 2023-01-03

**Authors:** Cheryl Cohen

**Affiliations:** 1 Centre for Respiratory Diseases and Meningitis, National Institute for Communicable Diseases, National Health Laboratory Service, Johannesburg, South Africa

This article addresses an important question of which settings are associated with most transmission of SARS-CoV-2 during lockdowns. This is relevant because findings can be used to develop more targeted guidance for lockdowns in future pandemics. The study leverage a very large dataset with carefully collected data on possible sources of infection acquisition outside the household. The analytic approach is appropriate and the conclusions are supported by the data. I have a few minor comments and suggestions.

 It would be nice to have a bit more information on the reasons for PCR testing in the cases ascertained on PCR through routine testing. I would also be interested to see whether the characteristics of cases ascertained on serology differed from those ascertained on PCR. Could you share the data on the proportion of cases who were symptomatic? It seems that much of the decision about who was an index case was around the time of onset of symptoms. Were the majority of cases symptomatic?

 A relatively large proportion of included cases were ascertained on serology. In this setting – these cases were only inferred as acquired outside the household if there was no other case in the household in the period of presumed infection (if I understood the methods correctly). This would lead to an increased likelihood to include people who did not transmit onward to household members. I.e. there may have been other household index cases serologically ascertained who would have been excluded because there was another case in the house and it was not possible to work out who was the index. Could this have introduced potential bias if single cases in the household differ from those who transmit onward?

Is the work clearly and accurately presented and does it cite the current literature?

Yes

If applicable, is the statistical analysis and its interpretation appropriate?

Yes

Are all the source data underlying the results available to ensure full reproducibility?

Yes

Is the study design appropriate and is the work technically sound?

Yes

Are the conclusions drawn adequately supported by the results?

Yes

Are sufficient details of methods and analysis provided to allow replication by others?

Yes

Reviewer Expertise:

Infectious disease epidemiology, respiratory disease burden, transmission and vaccine effectiveness

I confirm that I have read this submission and believe that I have an appropriate level of expertise to confirm that it is of an acceptable scientific standard.

# Peer review: Reviewer response for version 1

**DOI:** 10.21956/wellcomeopenres.19932.r53099

**Published:** 2022-11-28

**Authors:** Imen Ayouni Ep Labidi

**Affiliations:** 1 Department Paediatrics and Child Health, University of Cape Town, Cape Town, South Africa

I would like to thank the authors for the quality of their manuscript. The abstract is comprehensive and concise. It summarize the study and gives an understanding of the conducted research and findings.

 The introduction is very well developed and findings from previous research were summarized. The methods section is also very well developed and detailed especially the statistical methods. The results and discussion parts were very well elucidated and give a deep understanding of the study findings. 

 I would like the authors to consider the following minor comments:

1. Would you consider describing in details the different parts of the monthly questionnaire? 
2. In the discussion part, consider looking for studies that took into consideration personal hygiene practices and other non-pharmaceutical interventions other than lockdowns such as hand hygiene, mask wearing and distancing both in household and non-household transmission.

Is the work clearly and accurately presented and does it cite the current literature?

Yes

If applicable, is the statistical analysis and its interpretation appropriate?

I cannot comment. A qualified statistician is required.

Are all the source data underlying the results available to ensure full reproducibility?

Yes

Is the study design appropriate and is the work technically sound?

Yes

Are the conclusions drawn adequately supported by the results?

Yes

Are sufficient details of methods and analysis provided to allow replication by others?

Yes

Reviewer Expertise:

Vaccinology and maternal and child health

I confirm that I have read this submission and believe that I have an appropriate level of expertise to confirm that it is of an acceptable scientific standard.
